# Effects of Estradiol on Histological Parameters and Secretory Ability of Pituitary Mammotrophs in Ovariectomized Female Rats

**DOI:** 10.22074/cellj.2017.4334

**Published:** 2017-08-19

**Authors:** Nataša Ristić, Vladimir Ajdžanović, Milica Manojlović-Stojanoski, Jovana Maliković, Gordana Ušćebrka, Zorica Marković, Verica Milošević

**Affiliations:** 1 Department of Cytology, Institute for Biological Research "Siniša Stanković", University of Belgrade, Belgrade, Serbia; 2 Department of Veterinary Medicine, Faculty of Agriculture, University of Novi Sad, Novi Sad, Serbia; 3 Clinical Centre "Dr Dragiša Mišović", Belgrade, Serbia

**Keywords:** Mammotrophs, Prolactin, Estradiol, Ovariectomy, Rats

## Abstract

**Objective:**

Estrogen replacement therapy remains current as a therapeutic approach to treat menopausal symptoms and may significantly affect hormone-producing cells in the female pituitaries. The aim of this study was to examine the histological parameters of pituitary mammotrophs and prolactin secretion after chronic estradiol treatment in ovariectomized adult female rats, reflecting premature menopause.

**Materials and Methods:**

In this experimental study, adult female Wistar rats were divided into non-ovariectomized (C), ovariectomized (OVX) and estradiol-treated ovariectomized (OVX+E) groups. Estradiol dipropionate [0.625 mg/kg body mass per day] was administered for four weeks, while the C and OVX groups received vehicle alone. Mammotrophs were identified by the peroxidase-antiperoxidase (PAP) immunohistochemical procedure, while prolactin concentrations were measured by the non-isotopic two-step assay (Delfia) method. Comparison of the differences between groups was performed using one-way analysis of variance (ANOVA) and Tukay (honest significant difference) HSD test.

**Results:**

Ovariectomy caused significant (P<0.05) decreases in mammotroph optical density (OD), volume density (V_V_) and number per mm^2^ by 29, 27 and 34%, respectively, in comparison with the C females. In the OVX+E group, significant (P<0.05) increases in OD, cell volume, V_V_and number of mammotrophs per mm^2^ by 181, 15%, 5.8-fold and 5.2-fold, respectively, were observed when compared to OVX animals. The serum prolactin concentration in OVX females was significantly (P<0.05) decreased by 14% in comparison to the C group, while in OVX+E females, prolactin levels were significantly (P<0.05) increased by 53% compared to the OVX controls.

**Conclusion:**

Estradiol supplementation in ovariectomized females is followed by stimulatory histological and secretory changes of the mammotrophs. These results could serve as indicators of possible prolactinome development upon estradiol application in premature menopausal subjects.

## Introduction

Pituitary mammotrophs, i.e. prolactin(PRL) producing cells, in rats are distributed throughout the anterior-ventral part as well as in the areas near the gland pars intermedia (1). Classification of mammotrophs takes secretory granule size as a key criterion and accordingly three types of these cells are identified (1,2). PRL is a pulsatile secreted polypeptide hormone, functionally entwined with ovulation, pregnancy or nursing and characterized by a significant, age-related rise in females (1,3,4). Also, the number of female mammotrophs and their DNA content increase with ageing (5,6). Generally, the intensified PRL release with age is partly due to dysfunction of the dopaminergic mechanism in the hypothalamus (7). Studies in postmenopausal subjects have confirmed these secretory changes as far-reaching, since PRL levels do not decrease in that period of life (8). 

Premature menopause, linked with estrogen deficiency and infertility in young women, can develop spontaneously or may be caused by iatrogenic factors (9). A whole range of profound symptoms such as hot flashes, bone deterioration, decreased libido, cardiovascular issues and depression are included into its manifestation (9,10). When it comes to the mammotroph function in premature menopausal females, it was found long ago that PRL levels remain within normal range, while the pulsatility of secretion flattens (11). Estrogen replacement therapy is a common therapeutic approach to treat menopausal symptoms, with confirmed effectiveness in osteoporosis treatment (12), but due to possible adverse effects (breast or endometrial cancer, thromboembolic events) careful selection of dosage and duration of use are advised (13,15). 

Literature data have already pointed out that estradiol potently influences mammotroph differentiation, function and proliferation *in vitro* (16), while its effects in prematurely menopausal females remain insufficiently elaborated. Since estradiol application in menopausal subjects may change the histological parameters of pituitary mammotrophs and PRL secretion, our aim was to explain the phenomenology of potential estradiol-caused changes in ovariectomized adult female rats using modern histological and biochemical methodologies. Thus, in our model reflecting premature natural/iatrogenic menopause, we investigated the immunohistomorphometric characteristics of pituitary mammotrophs, using a design-based stereological approach, as well as their secretory ability, by measuring the optical density (OD) of immunostaining and the circulating PRL levels (17,18). 

## Materials and Methods

### Experimental design

In this experimental study, 21 female Wistar rats 12-weeks old (with the average body mass about 290 g) were housed in the experimental animal unit of the Institute for Biological Research "Siniša Stanković" and maintained under standard laboratory conditions (room temperature at 22 ± 2˚C and a 12 hour light: 12 hour dark cycle). Food and water were available ad libitum. The rats were divided into three groups (n=7). Females from two groups were bilaterally ovariectomized (OVX) under nembutal anesthesia (25 mg/ml distilled water). One month after ovariectomy, the first OVX group was treated through an intraperitoneal (i.p.) injection with 0.625 mg/kg b.m. of estradiol dipropionate (Oestradiol, Galenika a.d., Beograd, Srbija, OVX+E) per day for 4 weeks. The estradiol dose was selected based upon a previous work reporting its usage in clinical practice to treat postmenopausal women (19). Female rats in the second group represented long-term OVX controls injected with sterile olive oil for 4 weeks. The third group consisted of non-ovariectomized females injected with sterile olive oil (C) for 4 weeks. All females were sacrificed under ether anesthesia (ether ad narcosis Ph. Iug. III., Lek, Ljubljana, Slovenia) 24 hours after the last treatment. 

All animal procedures were adjusted to the European Communities Council Directive (86/609/EEC) and were approved by the Ethical Committee for the Use of Laboratory Animals of the Institute for Biological Research "Siniša Stanković", University of Belgrade, Serbia (Approval No. 2-12/13). 

#### Light microscopy and immunocytochemistry 

After decapitation, pituitary glands were excised, weighed and fixed in Bouin’s solution for 48 hours at room temperature. After dehydration in a series of increasing ethanol gradient, the pituitaries were enlightened in xylene and embedded in paraplast. Series of seven sections (5 mm) of the pituitary cut through three tissue levels (dorsal, middle and ventral portion) of the pars distalis were used for immunostaining. Mammotrophs were identified by immunohistochemistry using the peroxidase-antiperoxidase (PAP) method as previously described in detail (4). 

#### Morphometry

Image acquisition, morphometric assessment and digital imaging were performed using a light microscope (Olympus BX-51, Olympus, Japan) equipped with a microcator (Heidenhain MT1201, Heidenhain, USA) to control movements in the z-direction (0.2 µm accuracy), a motorized stage (Prior, Prior Scientifi Inc., USA) for stepwise displacement in the x-y direction (1 µm accuracy), and a CCD video camera (PixeLink, PixeLINK, Canada) connected to a 19” LCD computer monitor (Dell 1907FPc, Dell Inc., Round Rock, TX, USA). Image acquisition and stage movement were controlled by the newCAST stereological software package [Visiopharm Integrator System (VIS), version 2.12.1.0, Visiopharm, Denmark] running on a personal computer. 

Volume density (V_V_) represents the percentage of immunoreactive mammotrophs in the pituitary glands of experimental and control female rats. Two sections from the dorsal, three from the middle and two from the ventral part of rat pituitary glands were analyzed (the same sections were used in the subsequent estimation of number of mammotrophs per unit area-mm^2^ and cell volume determination). The counting area was defined using a mask tool. The new CAST software generated an interactive test grid, characterized by uniformly spaced test points for histomorphometric assessment. Test points hitting the immunoreactive mammotrophs and the uncolored phase of adenohypophysis were determined. V_Vs_ of mammotrophs were calculated as the ratio of the number of points hitting immunoreactive mammotrophs with nuclei divided by the number of points hitting the uncolored phase of adenohypophysis:

V_V_ (%)=P_p_/P_t_×100.

P_p_=points hitting the immunoreactive mammotrophs with nuclei, P_t_=points of the test system hitting the uncolored phase of adenohypophysis.

V_V_ of mammotrophs was calculated for each analyzed section. Then, the average value for seven analyzed sections was calculated and it represents the V_V_ of mammotrophs in a pituitary gland per animal.

The number of mammotrophs per mm^2^ was also calculated. In the first step, the areas of analyzed sections were determined by the Measure Properties option (Polygon area) and then, by simple point counting, the number of immunoreactive mammotrophs was estimated. Additionally, the number of mammotrophs was expressed per unit area (mm^2^). The single cell volume (μm3) of mammotrophs was measured using the rotator tool.

#### Pituitary mammotrophs optical density measurements

The Windows based ImageJ program (Image J, version 1.50f) was used for the analysis. Namely, 30 unbiasedly captured images (the microscopic tool has already been described: 2088×1550 pixels, ×63 objective magnification) per mammotroph specific-immunostained pituitary per animal were analyzed. Initially, the spectral deconvolution method of 3,3′-diaminobenzidine tetrahydrochloride (DAB)/Hematoxylin color spectra was performed, using optimized OD vectors of the color deconvolution plug-in for adequate separation of the DAB color spectra. To determine the OD for the red, green and blue (RGB) channel of Hematoxylin and DAB, we followed the protocol as previously described by Ruifrok and Johnston (20) and Varghese et al. (21). Since the OD is proportional to the concentration of the stain in mammotrophs, the amount of stain present is a factor determining the OD at a wave length specific to the stain, according to the formula:

OD=-log_10_ (I_C_/I_0.C_),

where I represents the transmitted light, I_C_ is the intensity of detected light after passing through a specimen and I_0.C_ refers to the intensity of light entering the specimen.

#### Hormonal analysis

Blood was collected from the trunk and separated sera samples of all animals were stored at the same time at -70˚C until assayed. Serum concentrations of PRL in control and experimental female rats were measured by the non-isotopic two-step assay (Delfia) method (hPRL-Delfia kits, LKB, Turku, Finland). 

#### Statistical analysis

Morphometric and hormonal data obtained for each group of female rats were averaged and SD was calculated with STATISTICA ^®^version 7.0 (StatSoft Inc., USA). One-way analysis of variance (ANOVA), followed by the multiple range test of Tukay (honest significant difference) HSD was used for comparison of the differences between groups. A probability value of 5% or less was considered statistically significant. 

### Results

The values of body mass, and absolute and relative pituitary weights are shown in Table 1. OVX caused significant (P<0.05) increase in the body mass of adult female rats by 16% in comparison with non-ovariectomized females, while absolute and relative pituitary weights were not significantly changed. In OVX+E females, body mass was significantly (P<0.05) decreased by 32% in comparison with the OVX group. Absolute and relative pituitary weights in the OVX+E group were 2.6-fold (P<0.05) and 3.4-fold (P<0.05) higher than in the OVX group, respectively. 

**Fig.1:**
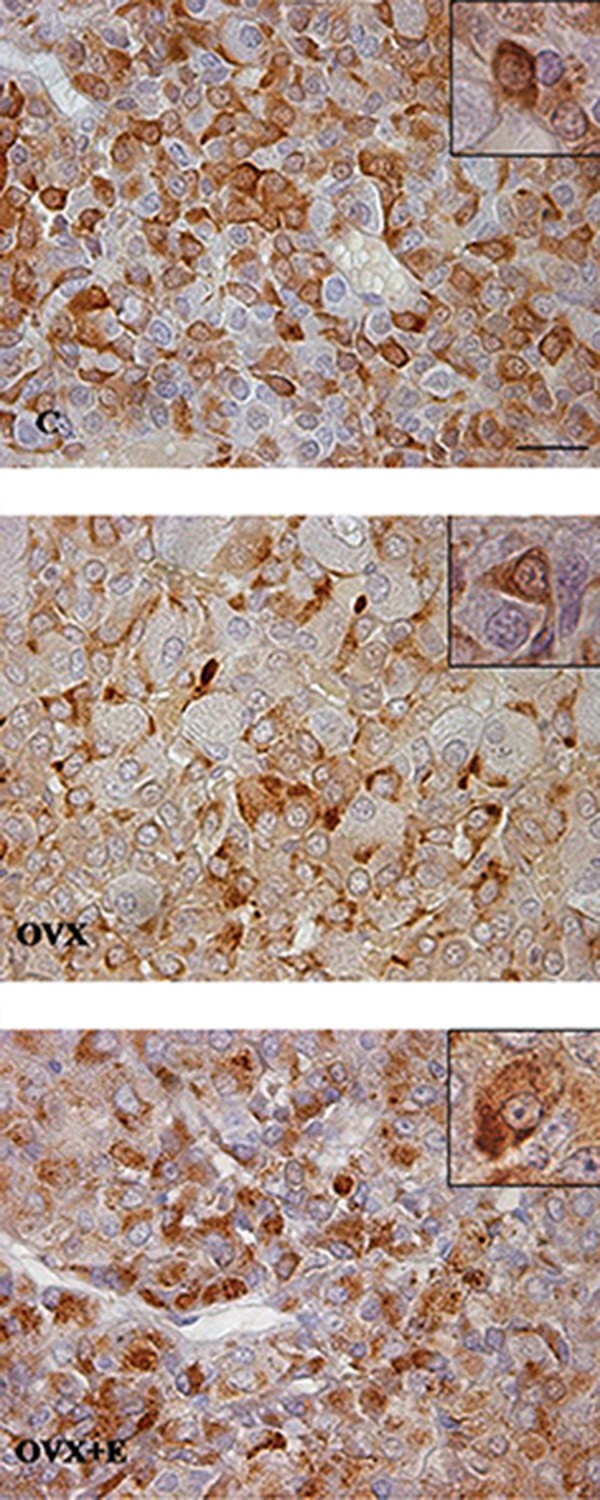
Immunopositive mammotrophs in pars distalis of the pituitary gland from C, OVX and OVX+E adult female rats (magnification: ×63, bar=16 μm). C; Non-ovariectomized, OVX; Ovariectomized, and OVX+E; Estradiol-treated ovariectomized.

In control, pituitary glands’ mammotrophs were spread throughout the pars distalis. These cells were oval or polygonal in shape and strong immunoreactivity was pronounced in their cytoplasm. After ovariectomy, mammotrophs were irregularly shaped and decreased intensity of immunostaining was noticed. In OVX+E females, the mammotrophs were irregularly shaped, with dark colored secretory granules (Fig .1). The quantitative analysis of mammotrophs showed that ovariectomy caused significant (P<0.05) decrease of their OD, V_V_ and number per mm^2^ by 29, 27 and 34%, respectively, in comparison with control non-ovariectomized females. In the OVX+E group, significant (P<0.05) increases in OD, cell volume, V_V_ and number of mammotrophs per mm^2^ by 181, 15%, 5.8-fold and 5.2-fold, respectively, were observed when compared to OVX animals (Figes.2, 3). The serum concentration of PRL in OVX females was significantly (P<0.05) decreased by 14% in comparison with nonovariectomized controls. In OVX females, estradiol treatment significantly (P<0.05) increased PRL concentration in serum by 53% compared to the OVX control (Fig .3).

**Table 1:** The body mass and absolute and relative pituitary weights among C, OVX and OVX+E adult female rats


Group	Body mass (g)	Absolute pituitary weight (mg)	Relative pituitary weight (mg%)

C	288 ± 18	14.2 ± 1.4	5.0 ± 0.7
OVX	334 ± 40^a^	16.5 ± 3.4	5.6 ± 1.3
OVX+E	227 ± 39^b^	42.8 ± 6.5^b^	18.8 ± 5.3^b^


Results are given as means ± SD (n=7). C; Non-ovariectomized, OVX; Ovariectomized, OVX+E; Estradiol-treated ovariectomized, ^a^; P<0.05 vs. C , ^b^; P<0.05 vs. OVX.

**Fig.2:**
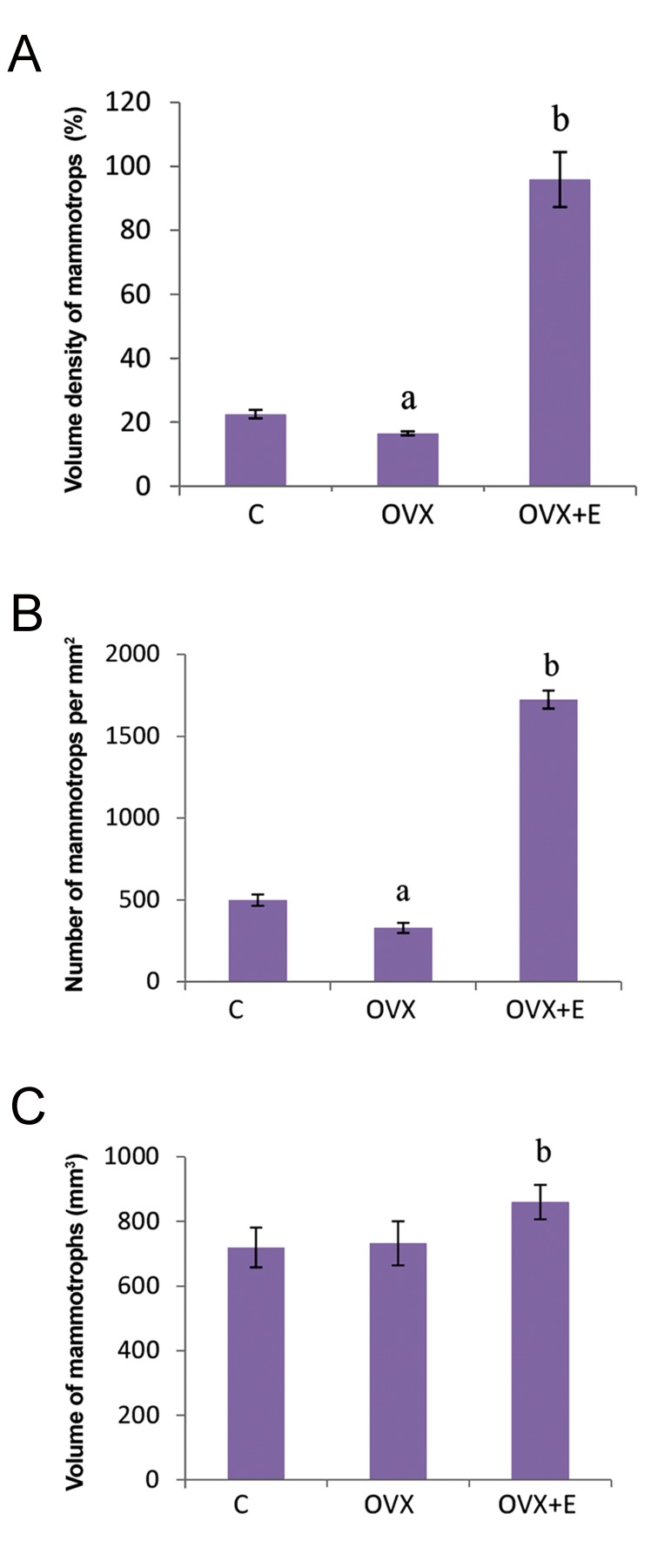
Morphometric parameters of pituitary mammotrophs. A. Vv (%), B. Number per mm^2^, and C. Volume (μm3) in C, OVX and OVX+E adult female rats, results are given as means ± SD (n=7). C; Non-ovariectomized, OVX; Ovariectomized, OVX+E; Estradioltreated ovariectomized, ^a^; P<0.05 vs. C, and ^b^; P<0.05 vs. OVX.

**Fig.3:**
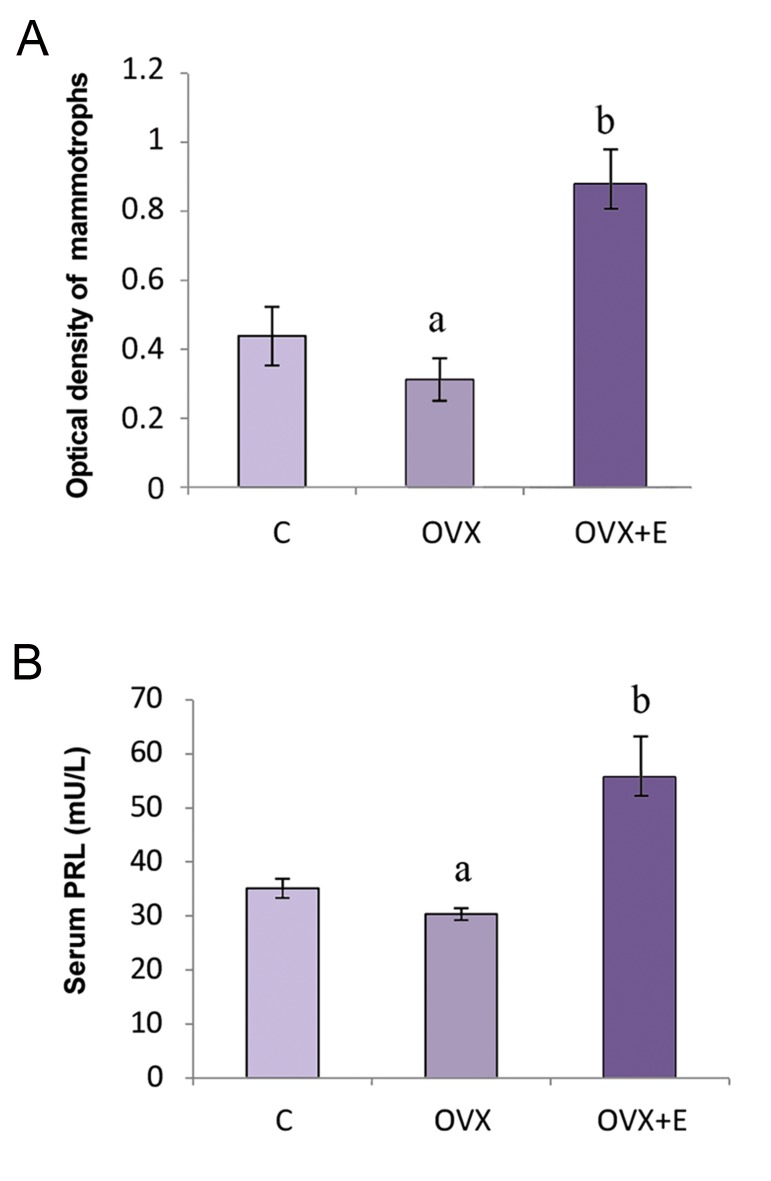
Secretory ability of pituitary mammotrophs. A. OD of pituitary mammotrophs and B. Serum PRL levels (mU/L) in C, OVX and OVX+E adult female rats, results are given as means ± SD (n=7). OD; Optical density, PRL; Prolactin, C; Non-ovariectomized, OVX; Ovariectomized, OVX+E; Estradiol-treated ovariectomized, ^a^; P<0.05 vs. C, and ^b^; P<0.05 vs. OVX.

## Discussion

Ovariectomized adult female rats, reflecting premature natural/iatrogenic menopause (17,18), were used in our experiment to investigate the histological parameters and secretory ability of their pituitary mammotrophs upon estradiol application. In brief, estradiol caused a significant increase in the number and size of mammotrophs, and positively affected the synthesis and secretion of PRL, based on OD and serum PRL levels determination. 

Firstly, we have provided evidence that hypoestrogenism, caused by ovariectomy, leads to an increase in body mass. Kurachi et al. (22) observed increased body mass upon ovariectomy accompanied by adipocyte hypertrophy. Estrogen withdrawal provoked by ovariectomy may influence body mass regulation at the central level, given the fact that estrogen receptors (ERs) α and β are found in the hypothalamic areas responsible for body mass regulation. In ERα deficient mice, a significant adipose tissue gain is observed, indicating a role of estrogen in the central regulation of body mass (23). Increases in the hypothalamic neuropeptide Y (NPY) expression (24) and decreases in hypothalamic corticotrophin- releasing hormone (CRH) immunoreactivity (25), both of which may promote hyperphagia, have also been established after ovariectomy. These changes in hypothalamic NPY and CRH could be explained by central leptin insensitivity associated with increased body mass in estrogen- deficient rats (26). Herein, chronic treatment of ovariectomized female rats with estradiol caused a significant body mass reduction in comparison to ovariectomy alone. Estrogen replacement therapy has already been shown to decrease body mass and food intake by the suppression of NPY or galanin- like peptidergic systems in the hypothalamic arcuate nucleus or medial preoptic area (27,28). Furthermore, estradiol treatment normalized all the changes in energy balance induced by ovariectomy, indicating that estrogen deficiency is responsible for energy imbalance (26). 

The further course of our study implied the examination of histological parameters of pituitary mammotrophs together with PRL levels measurement after ovariectomy and subsequent estradiol treatment. Mammotrophs generally show morphological, functional and physiological heterogeneity, the characteristics which are closely associated with estrogenic environment (29). Bearing in mind that the mammotroph cell population shows a remarkable ability to numerically change in response to various physiological and experimental conditions (30), their decreased V_V_ and number per mm^2^ in the hypoestrogenic milieu, caused by ovariectomy in our experiment, could be explained by the transdifferentiation of mammotrophs into gonadotropic cells. Transdifferentiation within the pituitary hormone-producing cell population is suggested to be an important event that aimed at providing homeostasis during specific physiological challenges (31). In line with the observed decrease in mammotroph immunohistomorphometric parameters and their potential transdifferentiation, serum concentrations of PRL expectedly fell in our study. Finally, the absolute and relative pituitary weights in our experimental set-up remain unchanged, which can also be supported by the gonadotropic cell population rise after transdifferentiation and some partially achieved equilibrium in this respect.

Chronic treatment of our ovariectomized female rats by estradiol led to an increase in OD values, individual cell volume, V_V_ and number of mammotrophs per mm^2^, which was followed by increased serum concentrations of PRL. The stimulatory effect of estradiol observed could be explained by the events at both the hypothalamic and the pituitary levels. Namely, at the level of hypothalamus, estrogen facilitates the release of hypophysiotrophic stimulatory factors (32), acting through widely present ERs in numerous hypothalamic neuronal populations (33). At the pituitary level, estrogen mediates the action of locally produced growth factors like insulin growth factor-1 (IGF-1), fibroblastic growth factor-2 (FGF-2) and epidermal growth factor (EGF) (34-36). In line with this, the increased values of stereological parameters (mammotroph volume and V_V_) observed are in coherence with promoted mammotroph cell synthetic activity as well as proliferation due to increased local production of FGF-2 in an estrogen environment (37). Based on the herein observed intensive mammotroph immunostaining/high OD, increased cell volume and elevated PRL levels, processes of PRL synthesis and secretion obviously remain under the stimulatory estradiol influence, as already reported (16). To note, PRL gene expression is enhanced through a mechanism that is mediated directly by the anterior pituitary ERs (38). Finally, estrogen promotes the mammotroph cell proliferation and early development of their hyperplasia by inducing Pit-1 transcription factor expression, which is considered the strongest predictor of prolactinomas (37, 39).

## Conclusion

The main contribution of our results to the investigation field is reflected in the detected ability of mammotrophs to change their appearance, size and secretion according to the varying estrogen environment, like ovariectomy and ovariectomy followed with estradiol treatment. By applying a modern histological approach, the mammotroph cell volume, V_V_, number of mammotrophs per mm^2^ and their OD were estimated, providing insight into the dynamics of mammotroph cell population in this respect. From the biomedical point of view, these quantitative and analytical histology based information could be relevant as an indicator of possible prolactinome or some other PRL-related disorder development upon estradiol application to premature menopausal subjects.
